# Catechol degradation on hematite/silica–gas interface as affected by gas composition and the formation of environmentally persistent free radicals

**DOI:** 10.1038/srep24494

**Published:** 2016-04-15

**Authors:** Hao Li, Huiying Guo, Bo Pan, Shaohua Liao, Di Zhang, Xikun Yang, Chungang Min, Baoshan Xing

**Affiliations:** 1 Faculty of Environmental Science & Engineering, Kunming University of Science & Technology, Kunming, 650500, P. R. China; 2 Research Center for Analysis and Measurement, Kunming University of Science and Technology, Kunming, 650093, P. R. China; 3 Stockbridge School of Agriculture, University of Massachusetts, Amherst, MA 01003, United States

## Abstract

Environmentally persistent free radicals (EPFRs) formed on a solid particle surface have received increasing attention because of their toxic effects. However, organic chemical fate regulated by EPFRs has rarely been investigated, and this information may provide the missing link in understanding their environmental behavior. Previous studies have suggested that the reduction of transition metals is involved in EPFRs formation. We thus hypothesize that an oxidative environment may inhibit EPFRs formation in particle-gas interface, which will consequently release free radicals and accelerate organic chemical degradation. Our result indicates that a 1% hematite coating on a silica surface inhibited catechol degradation in N_2_, especially at low catechol loadings on solid particles (*S*_CT_). However, under an O_2_ environment, catechol degradation decreased when *S*_CT_ was <1 μg/mg but increased when *S*_CT_ was >1 μg/mg. Stable organic free radicals were observed in the N_2_ system with g factors in the 2.0035–2.0050 range, suggesting the dominance of oxygen-centered free radicals. The introduction of O_2_ into the catechol degradation system substantially decreased the free radical signals and decreased the Fe(II) content. These results were observed in both dark and light irradiation systems, indicating the ubiquitous presence of EPFRs in regulating the fate of organic chemicals.

Iron oxides are ubiquitous in the environment and play important roles in geochemical processes. Because of the active chemical properties of iron, it can be chemically and biologically oxidized^1^ and reduced by sulfide^2^ and dissimilatory iron-reducing bacteria^3^. These iron-related processes are coupled to the carbon, oxygen and nitrogen cycles^4^.

The environmental fate and risks of various contaminants are also regulated by iron and iron oxides^5^. Fe(III) oxides with adsorbed Fe(II) can increase the transformation rate of various organic contaminants, such as nitrobenzenes^6^,^7^ and carbon tetrachloride^8^. Samples containing only Fe(III) oxyhydroxide in an aqueous phase or homogeneous Fe(II) solution does not exhibit reducing activity, and only samples with a solid-surface accumulation of Fe(II) rapidly reduced organic contaminants^9^,^10^. Although the mechanism is poorly understood, studies have emphasized the importance of adsorbed Fe(II)^11^. Based on isotope tracing studies, Handler *et al*. observed a near-complete exchange of Fe atoms between the aqueous phase and iron oxides but did not observe any change in the size and shape of the minerals^12^. In addition, interfacial electron transfer reactions were observed for goethite via current flowing through the bulk crystal. Fe(III) reduction and dissolution at one surface is coupled with Fe(II) oxidation and precipitation at another surface. Therefore, iron redox processes are also involved in organic contaminant transformation. More than that, these processes relate to inorganic pollutant behavior. For example, iron redox processes are intimately related to the fate of arsenic in the environment^13^. Fe(II) is a powerful reductant of highly toxic metals, such as Cr(VI)^14^ and U(VI)^15^. Fe(III)/Fe(II) redox cycles may cause decreased mobility (e.g., soluble Cr(VI) to sparingly soluble Cr(III)) or increased mobility (e.g., Hg(II) reduction to volatile Hg(0)) of contaminants^16^.

The above-mentioned studies were all carried out in aqueous phases. Recent studies have suggested that iron can form stable free radicals with organic molecules on the surface of solid particles in combustion process^17^,^18^. During this process, Fe(III) was reduced to Fe(II), and environmentally persistent free radicals (EPFRs) were formed. Studies have systematically provided evidence that EPFRs are more toxic than their parent compounds. EPFRs can cause pulmonary immunosuppression^19^, adverse infant respiratory health effects^20^, and decreased cardiac function^21^. The formation of EPFRs will affect the fate and risks of organic contaminants but the relevant research is limited. Our previous study suggested that after 3 h of photodegradation, catechol was partially protected by hematite in dry condition^22^. Based on electron paramagnetic resonance (EPR) measurements, we proposed that EPFR formation and Fe(III) reduction played a major role in this protection process.

Similar to aqueous phases, the Fe(II) on dry solid particles could easily be oxidized to Fe(III). However, the influence of oxic environment on the formation of EPFRs as well as organic contaminant degradation remains unknown. Therefore, this study was designed to provide dry and oxic conditions (pure O_2_) for Fe(II) oxidation in catechol degradation systems, which involved the formation of EPFRs. An anoxic environment (pure N_2_) and a mixed atmosphere were also studied for comparison. We hypothesize that an oxidative environment may inhibit EPFRs formation in particle-gas interface, which will consequently release free radicals and accelerate organic chemical degradation. In comparison to previous studies that primarily investigated formation mechanisms and health risks of EPFRs^18^,^20^, this study focused on the behavior of organic chemicals that are affected by EPFRs, which will improve our understanding of the fate and risk of organic contaminants and provide new insight into the role of iron redox cycling.

## Results

### Degradation of adsorbed catechol as affected by atmosphere composition

Catechol degradation was simulated under three different conditions as follows: in the dark for 3 d, light irradiation of >280 ± 10 nm for 180 min, or light irradiation of >340 ± 20 nm for 180 min. Catechol degradation as affected by atmosphere condition was similar in three light conditions (as summarized and compared in Fig. S3). Therefore, irradiation with >280 ± 10 nm light was the main focus of this study to enable a clear definition of the start and end of the catechol degradation reaction as well as a better comparison with previously reported literature results.

**Figure 1:**
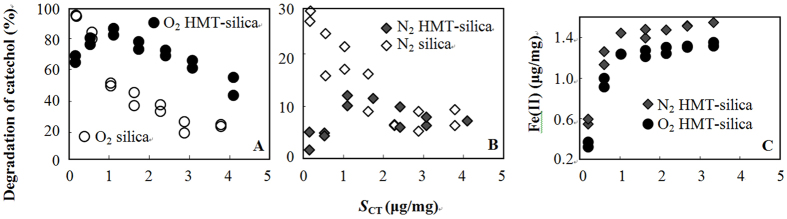
Degradation of catechol adsorbed on silica and HMT-silica under O_2_ (A) and N_2_ (B) after 180 min of UV/Vis light irradiation and the corresponding Fe(II) concentrations in the system (C). A comparison of the catechol degradation under O_2_ and N_2_ is shown in SI Fig. S3.

On both silica and HMT-silica, the presence of O_2 _substantially increased catechol degradation (Fig. 1A,B and SI Fig. S3). After 180 min of UV/Vis light irradiation, the catechol degradation on silica was approximately 100%, and the degradation on HMT-silica reached 90% under O_2_ (Fig. 1A) but was less than 30% under N_2_ (Fig. 1B). It should be noted that in this work, 100% N_2_ inflow may not repel the presence of O_2_ in the system, because of the O_2_ presence before N_2_ inflow and the surface adsorbed O_2_. Therefore, 100% N_2_ is referred to O_2_-limited atmosphere.

The degradation of catechol on silica decreased as the catechol solid-phase concentrations (*S*_CT_) increased (Figs 1A,B, or SI and S3). When different particles were compared under the same atmospheric conditions, the degradation of catechol exhibited a *S*_CT_ dependent behavior. At low *S*_CT_ (<0.5 μg/mg), the HMT coating on the silica surface decreased the degradation of catechol in N_2_ and O_2_ atmospheres (Fig. 1A,B). However, as *S*_CT_ increased to >1.0 μg/mg, the HMT coating exhibited different effects on the degradation of catechol in N_2_ and O_2_. The percent of degraded catechol increased after HMT coating in O_2_ (Fig. 1A) but was not substantially different from that of pure silica in N_2_ (Fig. 1B) at *S*_CT_ > 1.0 μg/mg.

**Figure 2:**
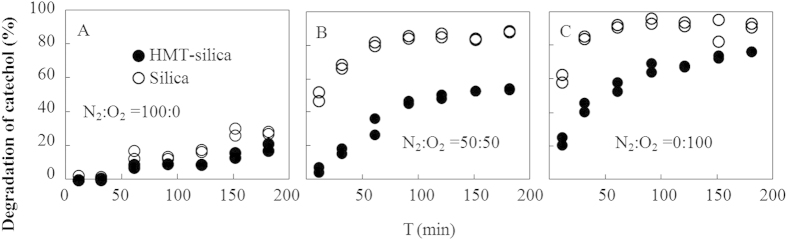
Catechol degradation kinetics at a solid-phase concentration of 0.11 ± 0.02 μg/mg in an N_2_:O_2_ atmosphere of 100:0 (A), 50:50 (B), and 0:100 (C). Catechol degradation kinetics at N_2_:O_2_ ratios of 30:70 and 70:30 are shown in SI Fig. S4. Catechol degradation kinetics at a solid-phase concentration of 2.10 ± 0.15 μg/mg in various N_2_:O_2_ ratios are shown in SI Fig. S5.

The catechol degradation kinetics were investigated for identical particles at five N_2_:O_2_ ratios (varied from 100:0 to 0:100). The *S*_CT_s were selected to cover both high and low concentration ranges (Fig. 1). In general, the degradation of catechol increased with increasing O_2_ concentrations. The degradation of catechol at *S*_CT_ = 2.10 ± 0.15 μg/mg continuously increased during the 180 min light irradiation (SI Fig. S5) but reached a plateau at *S*_CT_ = 0.11 ± 0.02 μg/mg within 180 min (Figs 2 and S4). However, the opposite effects were observed for HMT on catechol degradation at an *S*_CT_ of 0.11 ± 0.02 μg/mg (decreased degradation in the presence of HMT) and 2.10 ± 0.15 μg/mg (increased degradation in the presence of HMT).

### Free radical generation in the catechol degradation system

**Figure 3:**
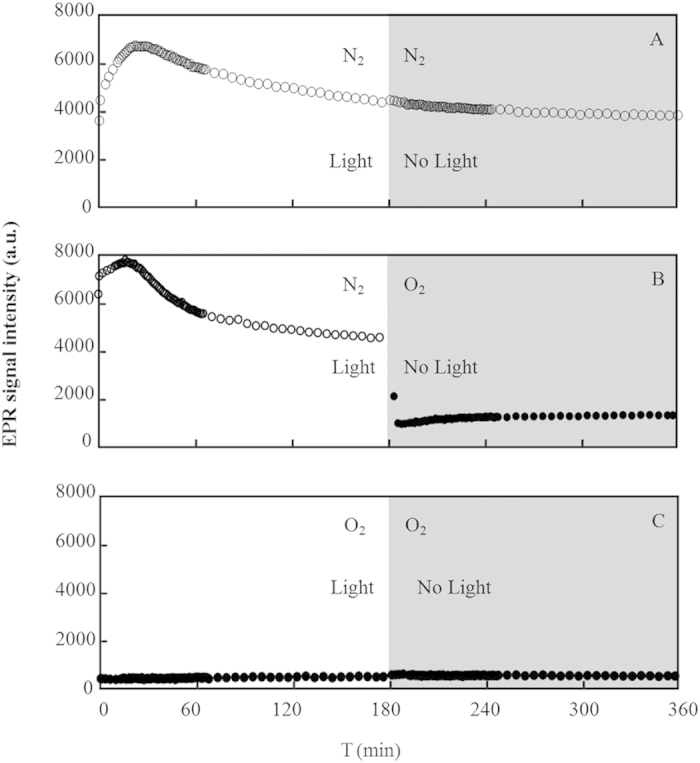
EPR signal intensity at *S*_CT _= 0.11 ± 0.02 μg/mg on HMT-silica. The gray background suggests the duration without light irradiation. EPR signals were collected in pure N_2_ (**A**), pure O_2_ (**C**), and pure N_2_ during light irradiation and then O_2_ after the light was turned off (**B**).

**Figure 4:**
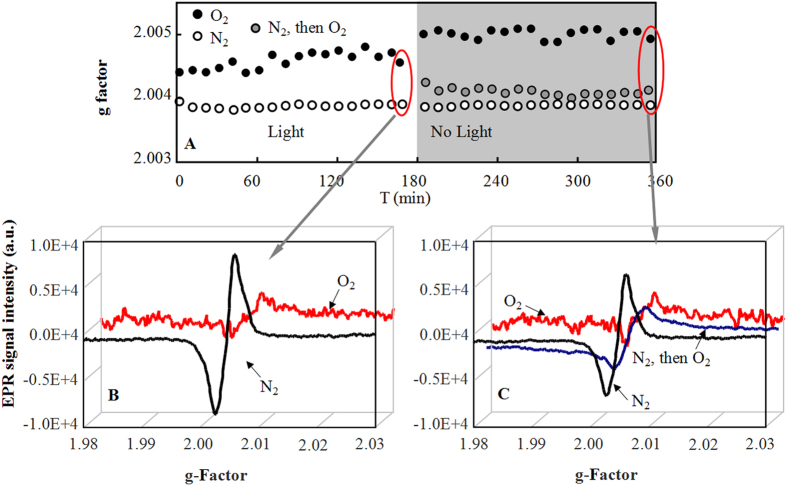
g factors (A) and typical EPR signals (B,C) at *S*_CT_ = 0.11 ± 0.02 μg/mg on HMT-silica. EPR signal intensities decreased in O_2_ compared to those in N_2_, which suggests the selective dissipation of free radicals with smaller g factors. The gray area represents the reaction after the light was turned off, and the gray circles represent the reactions in the system using pure N_2_ during light irradiation and then O_2_ after the light was turned off.

The EPR signals were selectively collected at *S*_CT_ = 2.10 ± 0.15 μg/mg and *S*_CT_ = 0.11 ± 0.02 μg/mg (Figs 3, 4, S6, S7, and S8). The two phenomena that were consistently observed in the EPR signal intensities for all of the systems were as follows: (1) The EPR signals were higher in the N_2_ systems than in the O_2_ systems, and (2) the EPR signals were higher in the silica systems than in the HMT-silica systems. For example, on HMT-silica, a considerable number of free radicals were generated in the pure N_2_ environment, and these free radicals remained after the light was turned off (Fig. 3A). However, in the pure O_2_ environment, the EPR signals on the HMT-silica exhibited a low intensity (Fig. 3C). In addition, when O_2_ was introduced after the light was turned off in the pure N_2_ environment, the EPR signals decreased substantially (Fig. 3B).

Although the EPR signal intensity variations in the presence of N_2_ and O_2_ were comparable to those in the HMT-silica systems, the g factors exhibited different trends (Figs 4 and S9). The g factors were typically higher than 2.0035, suggesting the dominance of the oxygen-centered free radicals. In the system with pure O_2_, the g factors were always higher than those in the pure N_2_ system. The results (2.0045–2.0050) fell into a range that is typical of semiquinone radical g factors^22^. The relatively lower g factors in the N_2_ system may suggest the presence of phenoxyl free radicals^23^. As shown in SI Figs S9 and S10, the g factors were relatively higher in the silica systems than those in in the HMT-silica systems. Similarly, when N_2_ was replaced with O_2_, a larger g factor increase was observed in the silica system than that in the HMT-silica system (Fig. S10).

### Fe(II) on HMT-silica and degradation products in the catechol degradation systems

Previous studies have proposed that the formation of EPFRs is accompanied by the reduction of Fe(III) to Fe(II)^18^,^22^. Therefore, this study employed two different methods to identify Fe(II) and Fe(III) in the reaction systems. One method involves the rapid dissolution of the solid particles in acid followed by trapping Fe(II) using PNL. The other method involves the direct measurement of the solid particles using XPS. Both methods detected a considerable amount of Fe(II) in the catechol degradation systems.

We detected a minimum amount of Fe(II) in the HMT-silica particles (without catechol) using PNL, which is most likely due to Fe(III) being transformed to Fe(II) in the acidic aqueous environment during Fe(II) measurement^24^. However, this amount of Fe(II) was an order of magnitude lower than the lowest Fe(II) amount detected in the catechol-involved systems, and this amount was considered the background quantity and was corrected.

Fe(II) was analyzed using the experimental setup as shown in Fig. 1A. After 180 min of light irradiation, the Fe(II) concentrations increased with *S*_CT_, suggesting that more Fe(II) reacted with catechol or its degradation by-products at higher *S*_CT_s. The Fe(II) concentrations in the O_2_–containing systems were lower than those in the N_2_–containing systems (Figs 1A and S11). The XPS measurements provided more direct evidence to support these observations. For example, (1) a large amount of Fe(II) was detected in the catechol degradation systems, and (2) a lower but still considerable amount of Fe(II) was observed in the O_2_ system compared to that in the N_2_ system. According to the XPS measurements, the surface Fe(II) accounted for 26.4–37.5% of the total Fe (Table 1 and SI Fig. S12).

The Fe(II) concentrations leveled off at approximately 1.3 μg/mg when the *S*_CT_ was higher than 1 μg/mg (Fig. 1C). This result was supported by the kinetics studies. The Fe(II) quickly reached a concentration of approximately 0.48 μg/mg at *S*_CT_ = 0.11 ±  ± 0.02 μg/mg, and no subsequent increase was observed (SI Fig. S11B).

**Figure 5:**
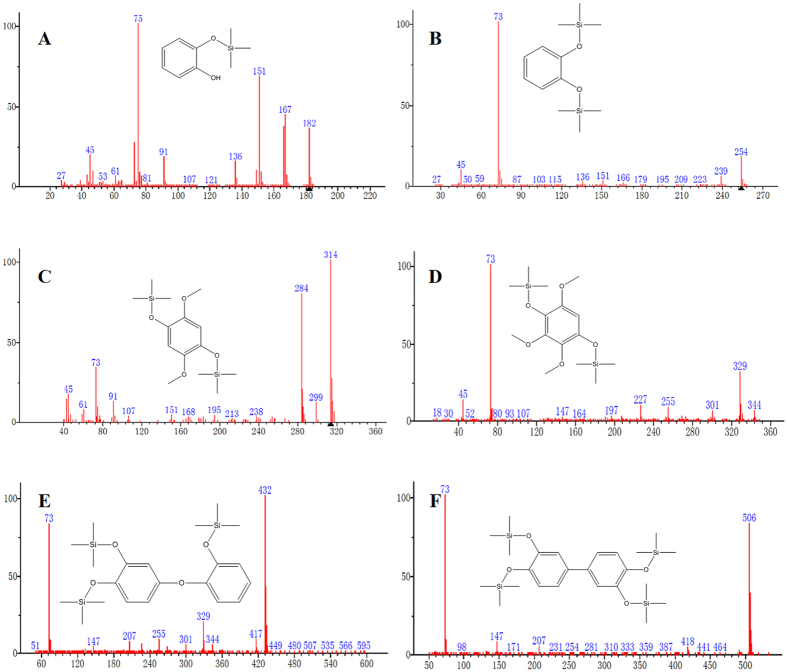
Major chemicals detected in the catechol degradation systems. Three groups of chemicals were typically observed included silylation products of catechol (panels (**A**,**B**) with retention times of 7.4 min and 9 min, respectively), catechol oxidation products (panels (**C**,**D**) with retention times of 12.2 min and 19.2 min, respectively) and dimers (panels (**E**,**F**) with retention times of 34.7 min and 35.6 min, respectively). The (CH_3_)_3_Si- resulted from the silylation.

Based on GC-MS, three groups of chemicals were typically observed during catechol degradation (Fig. 5). The first group consisted of the silylation products of catechol, such as 2-(trimethylsilyl)oxy-phenol (7.4 min retention time) and 1,2-benzenediol bis(trimethylsilyl) ether (9 min retention time). The second group of detected chemicals consisted of catechol oxidation products with two to three hydroxyl groups added to the benzene ring (i.e., 1,4-bis(trimethylsilyloxy)-2,5-dimethoxybenzene (12.2 min retention time) and 1,4-bis(trimethylsilyloxy)-2,5,6-trimethoxybenzene (19.2 min retention time)). The third group of detected chemicals consisted of catechol dimers. Both C-C (with *m*/*z* of 506) and C-O (with *m*/*z* of 434) coupled dimers were detected.

## Discussion

The relatively high catechol degradation in O_2_ than in N_2_ is not surprising because O_2_ provided an oxidative environment and accelerated catechol degradation. This result has also been observed in aqueous systems^25^. Since catechol degradation was observed both under light irradiation and in dark, catechol may degrade through photolysis and/or oxidation on particle surface. The decreasing degradation with increasing *S*_CT_ may be due to the catechol molecules being shielded from the light by the increased catechol loading on the surface of the particle, which results in *S*_CT_-dependent degradation kinetics.

We previously observed the stabilization of catechol on a HMT-silica surface but only in air without a clear insight into the role of the atmosphere^22^. The formation of EPFRs may explain the lower catechol degradation on HMT-silica compared to that on silica at a low *S*_CT_. In addition, the degradation increased with the *S*_CT_ in the HMT-silica systems when *S*_CT_ was less than 1 μg/mg (Fig. 1A). This relationship is most likely due to more effective catechol protection at a low *S*_CT_ (relatively higher Fe:catechol ratios), and therefore, the catechol degradation increased with increasing *S*_CT_.

In the catechol degradation kinetics experiments, the continuously increasing degradation at high *S*_CT_ (Fig. S5) but plateau at low *S*_CT_ (Fig. 2) were consistent with the earlier discussion whereby the degradation of catechol was slower at higher *S*_CT_s. The catechol degradation leveled off even when the degradation percentage was less than 50% in the HMT-silica system (Fig. 2A,B), suggesting that catechol was (to a certain extent) stabilized at a relatively low *S*_CT_.

The spin-spin interactions may have broadened the EPR lines under the O_2_ atmosphere, which may have reduced the EPR signals as shown in Fig. 3. However, the signals were not enhanced when the atmosphere was converted back to N_2_. Therefore, the reduced EPR signals were due to the O_2_ mediated quenching of free radicals. Based on the high degradation ratios in the O_2_ atmosphere, we propose that the intermediates with low g factors (likely phenoxyl free radicals) were degraded but those with high g factors remained.

The lower Fe(II) content detected in the O_2_ system may be due to the oxidation of Fe(II) by O_2_. However, this oxidation process altered only a small portion of Fe(II). Over 85% of the Fe(II) was detected in the O_2_ system. Fe(II) was detected after the light irradiation was turned off for over 180 min (liquid detection) and was stable for more than 3 days (solid particle detection) even in the presence of O_2_. In general, Fe(II) can be rapidly oxidized in oxic environments^26^. Our observation indicated that Fe(II) was stabilized in the reaction system.

Based on the low content of Fe(II) in the total Fe content (3–10%), the majority of the detected Fe(II) was located on the surface of the particle. The presence of Fe(II) in the O_2_ environment suggested that Fe(II) was stabilized on the particle surface, in relation to the protection of catechol. This result implies that the EPFRs were involved in decreasing catechol degradation.

The reason for the equilibrium Fe(II) concentration remains unknown but previous studies have reported similar Fe(III)/Fe(II) stoichiometric limits in Fe(II)/Fe(III) cycle related reactions. For example, Heijman *et al*. observed an accumulation of Fe(II) during the reduction of organic contaminants but the accumulation ended when one-third of the Fe(III) was reduced^27^. In addition, Gorski and Scherer reported that the oxidation of adsorbed Fe(II) was accompanied by the reduction of octahedral Fe(III) in magnetite to octahedral Fe(II), and Fe(II) uptake by these magnetite particles was limited by the stoichiometry of the magnetite particles^6^. Although the content of surface Fe(II) was too low to identify the magnetite using XRD, the XPS analysis yielded a surface Fe(II)/Fe(III) ratio of approximately 1:2 (Table 1), suggesting the possible formation of magnetite on the surface of HMT.

Correlating the catechol protection ratio to the Fe(II) content may not be appropriate because the oxidation and reduction of Fe are reversible in the reaction system. Additionally, the protection of catechol may not be permanent, and an equilibrium time point could not be determined to represent this correlation. However, a comparison of the molar concentrations is relatively easy. The Fe(II) content was higher than the stabilized catechol. Using HMT-silica at *S*_CT_ = 1 μg/mg as an example, the Fe(II) content was 2.4 and 8.8 times higher than the residual catechol content in N_2_ and O_2_, respectively. This result suggested that in addition to the dehydrogenated catechol radicals (catechol·)-Fe(II) complexes, other catechol degradation by-products may be involved in the formation of EPFRs.

The detected catechol in the degradation system was likely attributed to catechol not degraded, or transformed back to catechol from the (catechol·)-Fe(II) complexes^17^. The same three groups of degradation chemicals were detected in both the pure N_2_ and pure O_2_ environments, suggesting that the catechol degradation pathways were not substantially altered under different atmospheres. The abundance of catechol dimers was lower in the HMT-silica system than in the silica system. Previous studies have suggested that the polymerization of catechol is an important process for humic substance formation^28^ and could be promoted by transition metals^29^,^30^ in the aqueous phase. However, on the surface of a solid particle, the process may be different. The formation of EPFRs may have decreased the extent of catechol dimer formation. Based on the structure of the dimers, we speculate that both carbon-centered (as suggested by C-C coupling) and oxygen-centered (as suggested by C-O coupling) free radicals were formed in the catechol degradation system.

**Figure 6:**
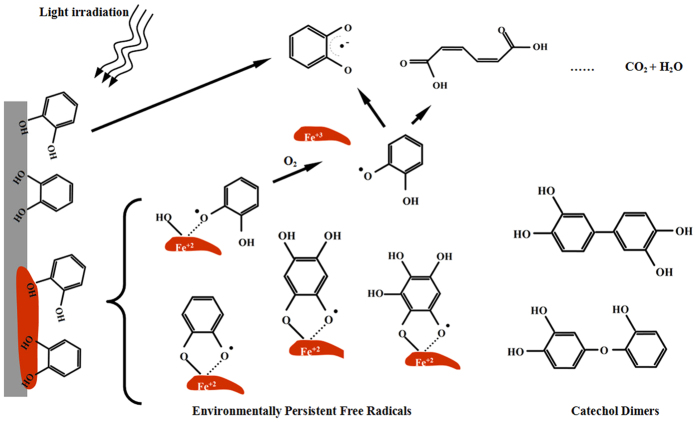
Degradation pathways of catechol under light irradiation. Environmentally persistent free radicals (EPFRs) form between Fe(II) and catechol or hydroxylated catechol. The introduction of O_2 _into the system releases phenoxyl free radicals and accelerates catechol degradation. Semiquinone free radicals complexed with Fe(II) are rather stable in O_2_. Catechol dimers are also formed in the catechol degradation system.

Based on all of our results, we propose the following reaction pathways for the degradation of catechol on silica and HMT-silica (Fig. 6). Under light irridiation and/or the presence of O_2_, catechol adsorbed on silica was firstly transformed into semiquinones. The benzene ring was then broken down, and catechol was ultimately degraded to CO_2_ and H_2_O. For the catechol adsorbed on HMT, EPFRs may form quickly. This process involves complexation between Fe(II) and the phenoxyl or semiquinone free radicals. With continuous light irradiation, a portion of the phenoxyl or semiquinone free radicals may degrade similar to that observed on the silica surface. In addition, catechol dimers were generated in both the silica and HMT-silica systems. In the presence of O_2_, a small fraction of Fe(II) was oxidized to Fe(III), and therefore, the phenoxyl free radicals were released, generating more free radicals in the system and accelerating catechol degradation. As the catechol degradation proceeded, more catechol formed semiquinone free radicals with Fe(II), which were more stable and resistant to oxidation. Therefore, both the catechol and Fe(II) were protected.

Although this discussion was focused on a system using UV/Vis light irradiation, the formation of EPFRs and their effect on catechol degradation were also observed for the dark system. For example, the following key phenomena were observed in the catechol auto-oxidation system in O_2_ or N_2_. (1) By comparing the silica and HMT-silica systems in O_2_, HMT protected catechol from degradation at low *S*_CT_s and accelerated catechol degradation at higher *S*_CT_s. These results were observed in both the 3-day degradation (SI Fig. S3) and degradation kinetics studies (SI Fig. S13). (2) A large amount of Fe(II) was detected in the catechol degradation systems, and the presence of O_2_ only partially oxidized Fe(II) (SI Fig. S14). (3) Higher EPR signals were observed in the silica systems compared to those for the HMT-silica systems, and the presence of O_2_ decreased the intensity of the EPR signals (SI Fig. S15).

A comparison of the results from the dark and light irradiated systems suggested that the formation of EPFRs may be involved in a wide range of organic chemical degradation systems. Although this EPFR formation may be ubiquitous in the environment, the effects of this process on chemical degradation are rarely investigated. Future studies in this area will provide new insights for organic chemical fate modeling and risk assessment.

## Methods

### Preparation of solid particles

Surrogate particles (1% Fe_2_O_3_ loaded on silica) were prepared using the incipient wetness impregnation method^18^. Briefly, silica gel particles were mixed with an iron nitrate nonahydrate (Fe(NO_3_)_3_·9H_2_O) solution, and the mixture was equilibrated for 24 h at room temperature. The resulting particles were freeze-dried and calcined at 450 °C in a muffle furnace with air for 5 h. The coated iron oxide particles consisted of hematite according to X-ray diffraction (XRD) (Supporting Information (SI) Fig. S1) and transmission electron microscopy coupled to X-ray energy dispersive spectrometry (TEM-EDX) (SI Fig. S2) measurements. These iron-coated particles are referred to as HMT-silica. Bare silica particles were also calcined using the same procedure whereby the iron nitrate nonahydrate solution was replaced by deionized water (Milli-Q water).

### Catechol loading on solid particles

Catechol is a common byproduct of organic compound degradation^31^ and ubiquitously present in the environment. Therefore, catechol was selected as the model chemical. This study focuses on the degradation of catechol adsorbed on solid particles. The catechol-loaded solid particles were prepared using a sorption freeze-dried process. Briefly, 1 mL of the catechol aqueous solution (with concentrations varying in the range of 100–1200 mg/L) and 300 mg of the particles were added to 8 mL amber glass vials. The loaded catechol concentration was determined as described in section 2.5 and expressed as the solid-phase concentration (*S*_CT_). This mixed solution was equilibrated for 12 h in the dark and then freeze-dried. The resulting solid particles were used in subsequent experiments. These particles were used within three days after preparation.

### Catechol degradation

Solid particles (80 mg) with different concentrations of catechol were poured into thin-wall quartz tubes (707-SQ-250M, Wilmad, USA). These particles were left in the dark for 3 days or subjected to light irradiation for 180 min in pure N_2_ or O_2_. The irradiation was provided by an arc light source (LOT-Oriel GmbH & CO. KG, Germany) equipped with a 100 W mercury lamp (USH-102D, Ushio, Japan). Two glass filters were used to restrict the emissions below 280 ± 10 nm or below 340 ± 20 nm (JB280 and JB340, Ceaulight, Beijing, China). The tubes were filled with a continuous flowing gas consisting of different N_2_:O_2_ ratios (100:0, 70:30, 50:50, 30:70, and 0:100). The gases (N_2_ and O_2_) were highly pure (≥99.999%), and the water content of the gases was less than 3 mg/m^3^. Because of the surface adsorbed O_2_ and O_2_ presence before N_2_ inflow, it is not practical to completely remove O_2_. Therefore, for the atomosphere condition of 100% N_2_, it accutually refers to O_2_-limited condition. As reference samples, solid particles were placed in the same atmosphere environment without UV/Vis light irradiation. The concentration of catechol was analyzed after 0, 10, 30, 60, 90, 120, 150, and 180 min of irradiation. All of the samples were run in duplicate.

### *In situ* EPR measurement

Solid particles using the same conditions as those in the catechol degradation experiment were analyzed on an EPR spectrometer (Bruker X-band A300-6/1) to identify free radicals. The typical operating parameters for all of the solid samples were as follows: a microwave frequency of 9.86 GHz, microwave power of 10 mW, center field of 3520 G, sweep width of 400 G, resolution of 1024 points, receiver gain of 3.17 × 10^3^, modulation frequency of 100 kHz, modulation amplitude of 3 G, time constant of 40.96 ms, and sweep time of 61.44 s. The relative intensity of the EPR response was measured as the peak-to-peak height. The light passed through the window shades of the resonant cavity and struck the particle samples to produce *in situ* light irradiation.

### Quantification of catechol

To analyze the concentration of catechol, the solid particles were extracted twice using 5 mL of methanol. According to the preliminary experiment, more than 95% of the applied catechol was recovered. The concentration of the catechol in the extract was determined using a high-performance liquid chromatograph (HPLC, Agilent Technologies 1200) equipped with an UV detector. The mobile phase was consisted of acetonitrile and deionized water (20:80 (v:v)), and the flow rate was 1 mL/min. The column was a reversed-phase C18 column (5 mm, 4.6 × 150 mm), and the wavelength for catechol detection was set to 220 nm. The concentration of catechol for the light irradiated particles was compared to that of the reference sample (particles without light irradiation), and the catechol degradation ratios determined.

### Fe characterization

The solid particles were also analyzed for Fe(III)/Fe(II) to investigate the reaction mechanisms. The Fe(II) concentration was determined using a 1,10-phenanthroline (PNL) spectrophotometric method because PNL forms a colored complex with Fe(II) (but not with Fe(III)) under weakly acidic conditions. To avoid Fe(II) oxidation by dissolved oxygen, all of the solutions used for Fe(II) analysis were purged for 10 min with N_2_. The solid particles were washed using 4 mL of HCl (3 mol/L) for 12 h with N_2 _purging. Two milliliters of the liquid were transferred to a 25 mL colorimetric tube. Then, 6 mL of the sodium acetate buffer and 2 mL of PNL were added. The solutions were shaken and reacted for 10 min followed by analysis on a UV spectrophotometer (UV-2600, Shimadzu) at 510 nm to determine the Fe(II) concentration.

The silica particles as well as the particles coated with iron were characterized using both X-ray diffraction (XRD) and X-ray photoelectron spectroscopy (XPS). The XRD patterns were recorded using an X-ray diffractometer (D/Max-2200, Rigaku, Japan) with a Cu Kα radiation source (λ = 0.15406 nm) operated at 40 kV and 40 mA. The XPS analyses were performed on a PHI5000 Versaprobe-II Scanning XPS Microprobe system. This system employed an Al Kα X-ray radiation source with energy from an Al rake at a power of 50 W, and the pass energy was 46.95 eV. The binding energy (BE) was relative to the adventitious C1 s line at 284.8 eV.

### GC-MS analyses

The catechol degradation samples were selectively analyzed on a gas chromatograph-mass spectrometer (GC-MS, Agilent Technologies, model 7890A-5975C). The methanol extracts of the solid particles were dried under N_2_ flux. The dried residues were silylated by adding 100 μL of a commercial mixture of N,O,-bis(trimethylsilyl)trifluoracetamide and trimethylchlorosilane ( BSTFA/TMCS 99:1 (Alfa Aesar)), which was allowed to react at 60 °C for 15 min. The derivatized samples (1 μL) were injected into a GC-MS equipped with an HP-5 MS capillary column (30 m long, 0.25 mm i.d., and 0.25-μm film thickness). The column oven was programmed with an initial temperature of 80 °C for 1 min followed by a gradient from 80–180 °C at 4 °C/min, 180–320 °C at 10 °C /min, and a final temperature of 320 °C, which was maintained for 10 min. The MS was operated in a range from m/z 50 to 1000 with electron impact ionization energy of 70 eV. A pure catechol solution was also silylated and analyzed using the same procedure to interpret the spectral data. The mass spectral library of the National Institute of Standards and Technology (NIST 11) was used to qualitatively identify the molecular products.

## Additional Information

**How to cite this article**: Li, H. *et al*. Catechol degradation on hematite/silica–gas interface as affected by gas composition and the formation of environmentally persistent free radicals. *Sci. Rep.* **6**, 24494; doi: 10.1038/srep24494 (2016).

## Supplementary Material

Supplementary Information

## Figures and Tables

**Table 1:** Surface elemental analysis using XPS.

*S*_CT_(μg/mg)	Sample	C	O	N	Si	Fe	Fe(II)	Fe(III)	Fe(II)%	Fe(III)%
0	silica	2.22	68.4	0.28	29.1	–	–	–	–	–
	HMT+silica	1.91	68.6	0.25	28.8	0.48	–	0.48	–	100
0.11 ± 0.02	silica N_2_	1.53	68.8	0.51	29.2	–	–	–	–	–
	silica O_2_	1.44	68.6	0.43	29.5	–	–	–	–	–
	HMT+silica N_2_	1.70	68.9	0.26	28.7	0.43	0.15	0.28	35.1	64.9
	HMT+silica O_2_	1.59	68.5	0.59	29.0	0.37	0.11	0.26	30.1	70.0
2.10 ± 0.15	silica N_2_	2.56	68.1	0.31	29.1	–	–	–	–	–
	silica O_2_	2.10	69.0	0.28	28.7	–	–	–	–	–
	HMT+silica N_2_	2.19	68.2	0.49	28.7	0.42	0.16	0.26	37.5	62.6
	HMT+silica O_2_	1.43	69.3	0.29	28.6	0.41	0.11	0.30	26.4	73.6
