# Supplementary material for: Catechol degradation on hematite/silica–gas interface as affected by gas composition and the formation of environmentally persistent free radicals
Source: Sci Rep. 2016 Apr 15;6:24494. doi: 10.1038/srep24494 (PMC4832247; doi:10.1038/srep24494)
Supplement: Supplementary Information [file srep24494-s1.doc]

**Supporting Information**

**Catechol degradation on hematite/silica – gas interface as affected by gas composition and the formation of environmentally persistent free radicals**

Hao Li^1^, Huiying Guo^1^, Bo Pan^1^*, Shaohua Liao^1^, Di Zhang^1^, Xikun Yang^3^, Chungang Min^3^, and Baoshan Xing^2^*

1. Faculty of Environmental Science & Engineering, Kunming University of Science & Technology, Kunming 650500, P. R. China

2. Stockbridge School of Agriculture, University of Massachusetts, Amherst, MA 01003, United States

3. Research Center for Analysis and Measurement, Kunming University of Science and Technology, Kunming 650093, P. R. China

Corresponding authors: Bo Pan, phone: 86-871-65102829, e-mail: panbocai@gmail.com; Baoshan Xing, phone: 413-545-5212, e-mail: [bx@umass.edu](mailto:bx@umass.edu)

Number of pages: 15

Number of figures: 16

XRD Intensity (a.u.)

20

80

60

40

2θ/degree

Silica

HMT-silica

Pure HMT

Figure S1. XRD patterns of silica and HMT-silica particles. The symbols of indicate the typical XRD peaks of HMT. Pure HMT was generated using the same method for HMT-silica.

0

1000

2000

3000

0

10000

20000

30000

40000

50000

0

1000

2000

3000

-5

0

5

10

15

20

25


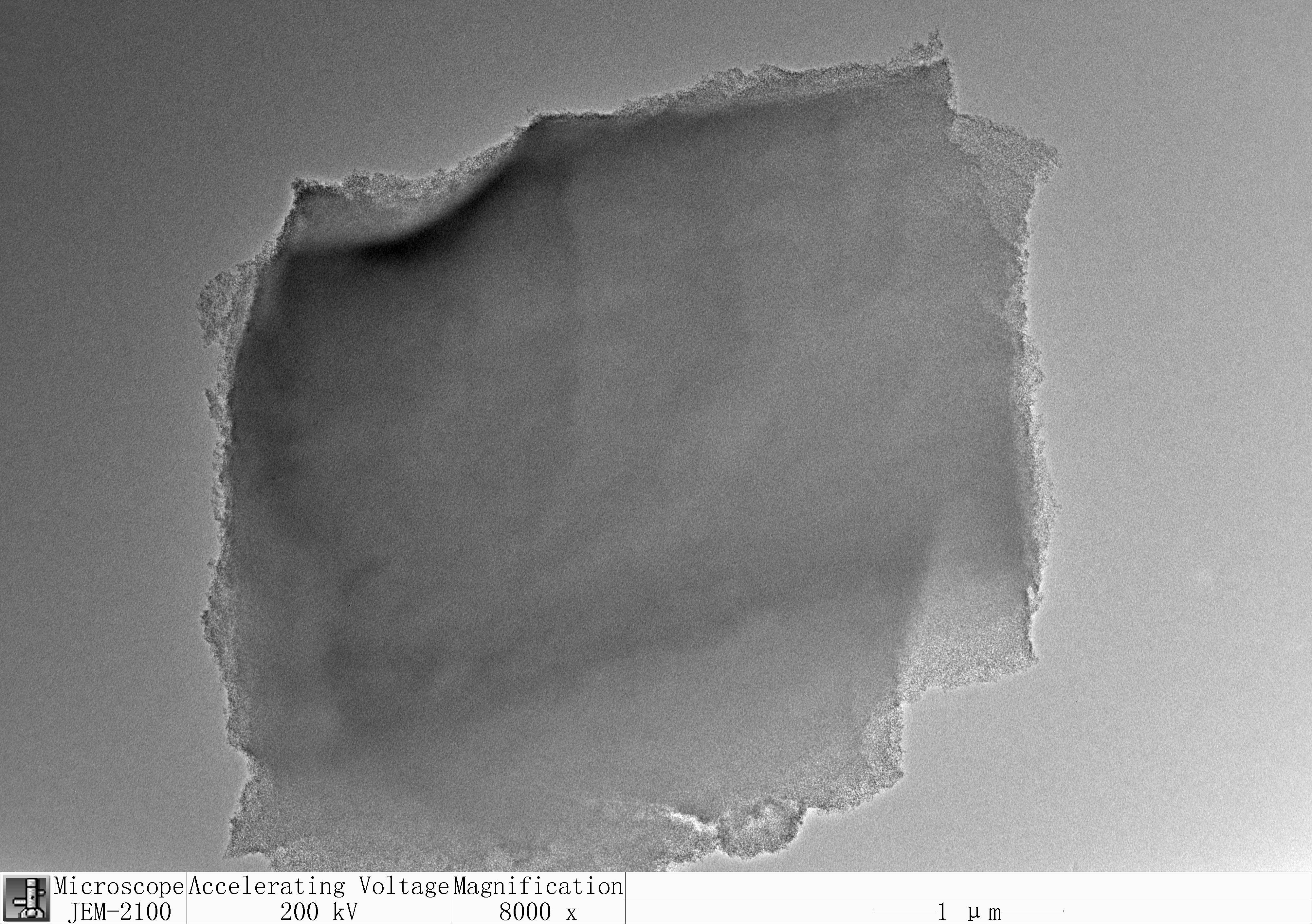

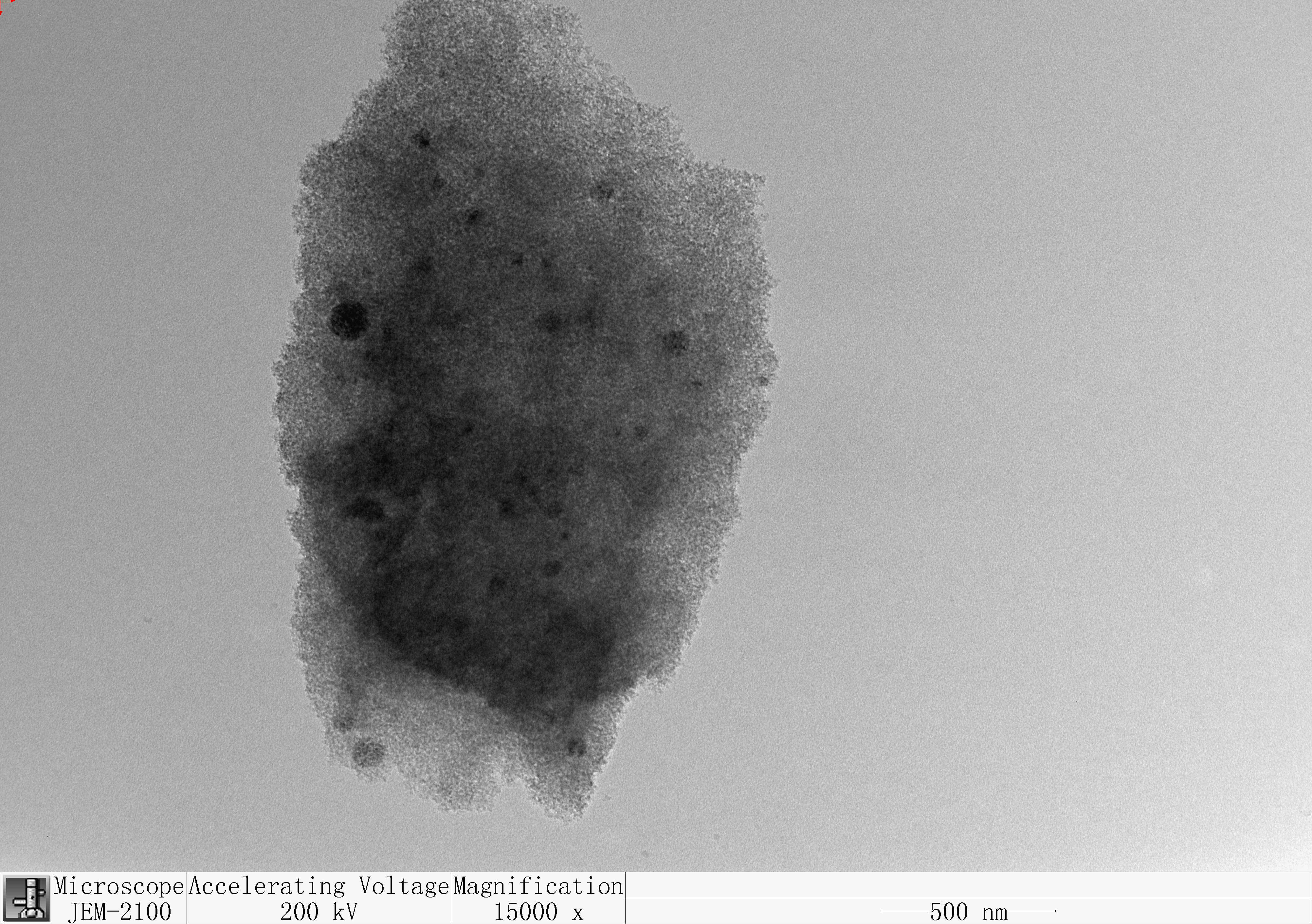

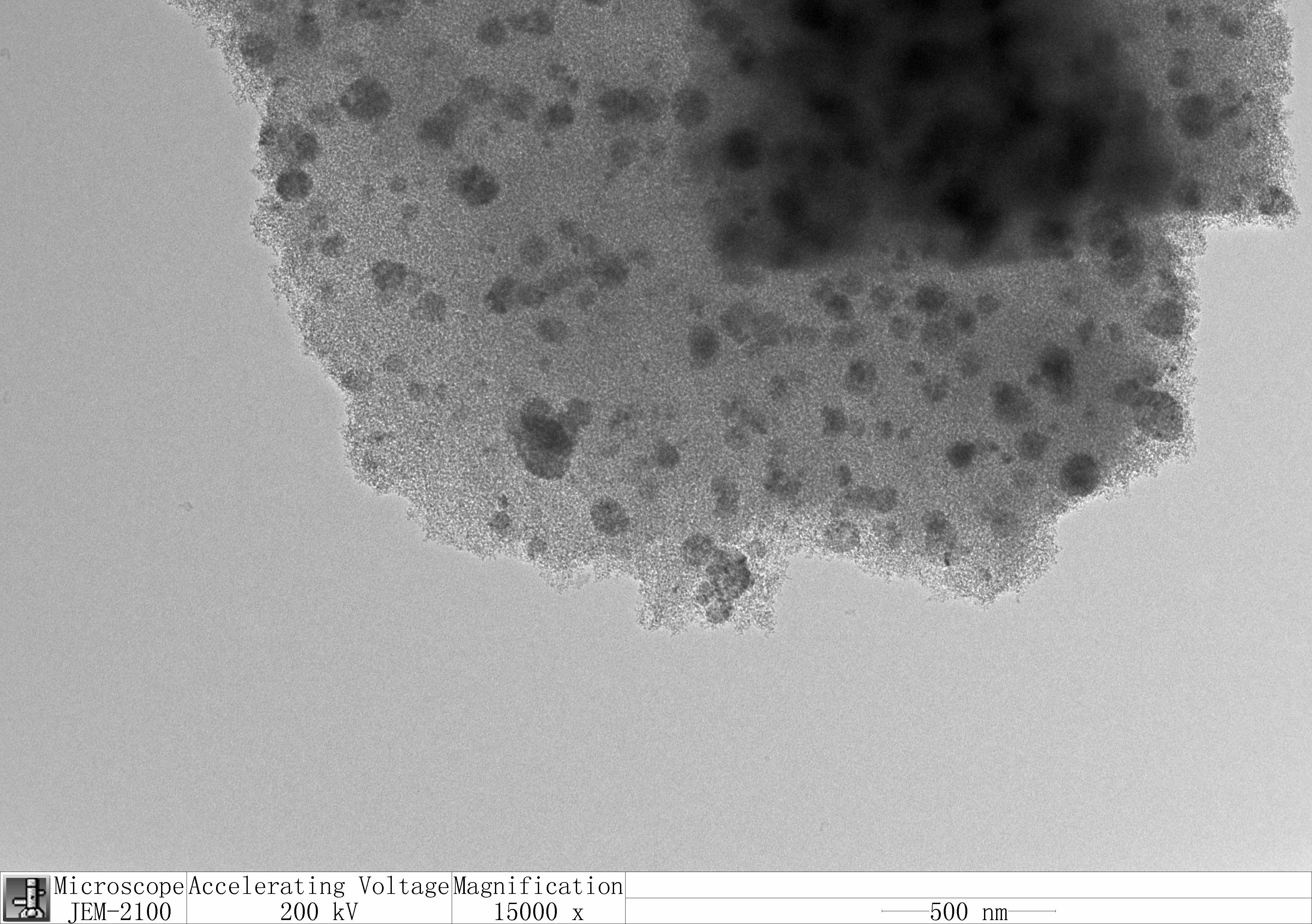


Si

O

Cu

Cu

Cu

Si

O

Cu

Cu

Fe

Cu

Fe

Fe

Cu

Cu

Fe

Fe

Si

O

Fe

ElementWeight%O K41.8Si K41.6Fe K6.4Cu K10.3

1 μm

ElementWeight%O K29.4Si K33.2Fe K0.34Cu K36.9

ElementWeight%O K49.8Si K44.4Cu K5.8

1 μm

1 μm

keV

Counts

**A**

**B**

**C**

Figure S2. The TEM-EDX analysis of solid particles. Silica, 1% HMT-silica and 10% HMT-silica particles were separately presented in panels A, B, and C, respectively. The energy-dispersive X-ray spectroscopy (EDX) of the solid particles on Cu foils were investigated by transmission electron microscopy (TEM, JEM 2100) with accelerating voltage of 200 kV. Chemical composition of the composites was semi-quantitatively analyzed using EDX data. The collection time for EDX data was 50 s.

0

30

60

90

0

1

2

3

4

5

0

20

40

60

80

100

0

1

2

3

4

5

**Degradation of catechol (%)**

***S*_CT_ (μg/mg)**

O_2_ HMT-silica

N_2_ HMT-silica

**A**

O_2_ silica

N_2_ silica

**B**

0

20

40

60

80

0.0

1.0

2.0

3.0

4.0

0

20

40

60

80

100

0.0

1.0

2.0

3.0

4.0

**Degradation of catechol (%)**

***S*_CT_ (μg/mg)**

**C**

**D**

0

20

40

60

80

100

0.0

1.0

2.0

3.0

0

20

40

60

80

100

0.0

1.0

2.0

3.0

**Degradation of catechol (%)**

***S*_CT_ (μg/mg)**

**E**

**F**

0

20

40

60

80

100

0

1

2

3

4

5

HMT-Silica O_2_

Silica O_2_

HMT-Silica N_2_

Silica N_2_

**Degradation of catechol (%)**

**G**

***S*_CT_ (μg/mg)**

0

20

40

60

80

100

0

1

2

3

4

**Degradation of catechol (%)**

HMT-Silica O_2_

Silica O_2_

HMT-Silica N_2_

Silica N_2_

**H**

***S*_CT_ (μg/mg)**

0

20

40

60

80

100

0

1

2

3

HMT-Silica O_2_

Silica O_2_

HMT-Silica N_2_

Silica N_2_

**Degradation of catechol (%)**

***S*_CT_ (μg/mg)**

**I**

Figure S3. The degradation ratios of catechol adsorbed on HMT-silica (A, C, and E) and silica (B, D and F) in O_2_ and N_2_ after 180-min UV-light irradiation of > 280 nm (A and B), after 180-min UV-light irradiation of > 340 nm (C and D), and after 3 d reaction in the dark (E and F). Panel G is the combination of A and B, panel H is the combination of C and D, and panel I of E and F. Comparing silica and HMT-silica systems in O_2_, HMT protected catechol from degradation at low *S*_CT_, but accelerated catechol degradation at high *S*_CT_.

0

20

40

60

80

100

0

50

100

150

200

0

20

40

60

80

100

0

50

100

150

200

N_2_:O_2_ =30:70

T (min)

HMT-silica

Silica

**Degradation of catechol (%)**

N_2_:O_2_ =70:30

**A**

**B**

Figure S4. Catechol degradation at solid-phase concentration of 0.11 ± 0.02 μg/mg in the atmosphere of N_2_:O_2_ at 30:70 (A) and 70:30 (B).

0

20

40

60

80

100

0

50

100

150

200

T (min)

HMT-silica

Silica

50

100

150

200

50

100

150

200

N_2_:O_2_ =100:0

N_2_:O_2_ =50:50

N_2_:O_2_ =0:100

A

B

C

**Degradation of catechol (%)**

0

20

40

60

80

100

0

50

100

150

200

0

50

100

150

200

0

20

40

60

80

100

T (min)

N_2_:O_2_ =30:70

HMT-silica

Silica

N_2_:O_2_ =70:30

**D**

**E**

**Degradation of catechol (%)**

Figure S5. Catechol degradation kinetics at solid-phase concentration of 2.10 ± 0.15 μg/mg in the atmosphere of N_2_:O_2_ at 100:0 (A), 50:50 (B), 0:100 (C), 30:70 (D) and 70:30 (E).

0

3000

6000

9000

12000

0

60

120

180

240

300

360

No UV

UV

N_2_

N_2_

No UV

UV

N_2_

O_2_

No UV

UV

O_2_

O_2_

T (min)

0

3000

6000

9000

12000

0

3000

6000

9000

12000

**EPR signal intensity (a.u.)**

Figure S6. EPR signal intensity at *S*_CT_ = 2.10 ± 0.15 μg/mg on HMT-silica. The grey background suggests the duration without UV light irradiation. EPR signals were collected in pure N_2_ (A) or pure O_2_ (C) as well as the system with pure N_2_ during UV light irradiation and then was replaced by O_2_ after the UV light was turned off (B).

0

6000

12000

18000

0

60

120

180

240

300

360

No UV

UV

N_2_

N_2_

No UV

UV

N_2_

O_2_

No UV

UV

O_2_

O_2_

T (min)

0

6000

12000

18000

0

6000

12000

18000

**EPR signal intensity (a.u.)**

Figure S7. EPR signal intensity at *S*_CT_ = 2.10 ± 0.15 μg/mg on silica. The grey background suggests the duration without UV light irradiation. EPR signals were collected in pure N_2_ (A) or pure O_2_ (C) as well as the system with pure N_2_ during UV light irradiation and then was replaced by O_2_ after the UV light was turned off (B).

0

4000

8000

12000

16000

0

60

120

180

240

300

360

No UV

UV

N_2_

N_2_

No UV

UV

N_2_

O_2_

No UV

UV

O_2_

O_2_

T (min)

0

4000

8000

12000

16000

0

4000

8000

12000

16000

**EPR signal intensity (a.u.)**

Figure S8. EPR signal intensity at *S*_CT_ = 0.11 ± 0.02 μg/mg on silica. The grey background suggests the duration without UV light irradiation. EPR signals were collected in pure N_2_ (A) or pure O_2_ (C) as well as the system with pure N_2_ during UV light irradiation and then was replaced by O_2_ after the UV light was turned off (B).

0

60

120

180

240

300

360

T (min)

**g Factor**

2.003

2.004

2.005

No UV

UV

N_2_ HMT-silica

O_2_ HMT-silica

No UV

UV

N_2_ silica

O_2_ silica

2.003

2.004

2.005

Figure S9. g values of EPR signals at *S*_CT_ = 2.10 ± 0.15 μg/mg on silica and HMT-silica.

0

60

120

180

240

300

360

T (min)

**g factor**

2.003

2.004

2.005

No UV

UV

N_2_ HMT-silica

O_2_ HMT-silica

No UV

UV

N_2_ silica

O_2_ silica

2.003

2.004

2.005

Figure S10. g values of EPR signals at solid-phase concentration 0.11 ± 0.02 μg/mg on silica and HMT-silica.

0.4

0.8

1.2

0

20

40

60

80

**Fe(II) (μg/mg)**

**T (min)**

0.3

0.4

0.5

0.6

0

50

100

150

200

**T (min)**

N_2_ HMT-silica

O_2_ HMT-silica

**B**

**A**

N_2_ HMT-silica

O_2_ HMT-silica

Figure S11. Fe(II) formation kinetics at *S*_CT_ = 2.10 ± 0.15 μg/mg (A) and 0.11 ± 0.02 μg/mg (B) on HMT-silica in N_2_ and O_2_.

Figure S12. Typical XPS analysis of Fe species on HMT-silica after catechol degradation. Both spectrum were recorded at *S*_CT_ = 2.10 ± 0.15 μg/mg in N_2_ (A) or O_2_ (B).

0

20

40

60

80

100

0

20

40

60

80

100

Silica

HMT

0

20

40

60

80

100

Time (h)

0

20

40

60

80

100

0.14±0.02

0.55±0.06

0.98±0.08

Silica

HMT

Silica

HMT

A

**B**

C

**Degradation of catechol (%)**

Figure S13. Catechol degradation kinetics under O_2_ in dark systems. HMT protected catechol from degradation at low *S*_CT_, but accelerated catechol degradation at high *S*_CT_.

0.0

0.2

0.4

0.6

0.8

1.0

0.0

0.5

1.0

1.5

2.0

2.5

3.0

Fe^2+^ (μg/mg)

*S*_e_ (μg/mg)

HMT-O_2_

HMT-N_2_

Figure S14. Fe(II) in HMT-silica after catechol degradation in dark system for 3 d. Significant amount of Fe(II) was detected in catechol degradation systems, and the presence of O_2_ only partially oxidized Fe(II).

Silica-N_2_

Silica-O_2_

0

4

8

12

16

Time (h)

HMT-N_2_

HMT-O_2_

0

5

10

15

20

25

30

35

0

5

10

15

20

25

0

20

40

60

80

0

5

10

15

20

25

0

20

40

60

80

0

5

10

15

20

25

30

35

0

20

40

60

80

0

4

8

12

16

EPR signal intensity (×10^3^a.u.)

Figure S15. EPR signals after catechol degradation in dark systems. Stronger EPR signals were observed in silica systems than in HMT-silica systems and the presence of O_2_ significantly decreased EPR signals.
